# Evaluation of Direct Oral Anticoagulant Reversal Agents in Intracranial Hemorrhage: A Systematic Review and Meta-analysis

**DOI:** 10.1001/jamanetworkopen.2022.40145

**Published:** 2022-11-04

**Authors:** Rahul Chaudhary, Amteshwar Singh, Rohit Chaudhary, Michael Bashline, Damon E. Houghton, Alejandro Rabinstein, Jill Adamski, Richard Arndt, Narith N. Ou, Maria I. Rudis, Caitlin S. Brown, Erin D. Wieruszewski, Matthew Wanek, Nathan J. Brinkman, Jane A. Linderbaum, Melissa A. Sorenson, John L. Atkinson, Kristine M. Thompson, Aryan N. Aiyer, Robert D. McBane

**Affiliations:** 1 Division of Cardiology, Department of Medicine, University of Pittsburgh Heart and Vascular Institute, Pittsburgh, Pennsylvania; 2 Division of Hospital Medicine, Department of Medicine, Johns Hopkins University School of Medicine, Baltimore, Maryland; 3 Deakin University, Melbourne, Australia; 4 Division of Vascular Medicine, Department of Cardiovascular Diseases, Mayo Clinic Rochester, Minnesota; 5 Division of Hematology/Oncology, Department of Internal Medicine, Mayo Clinic Rochester, Minnesota; 6 Department of Neurology, Mayo Clinic Rochester, Minnesota; 7 Department of Laboratory Medicine and Pathology, Mayo Clinic Hospital, Phoenix, Arizona; 8 Department of Pharmacy, Mayo Clinic Health System, Eau Claire, Wisconsin; 9 Department of Pharmacy, Mayo Clinic, Rochester, Minnesota; 10 Departments of Pharmacy and Emergency Medicine, Mayo Clinic, Rochester, Minnesota; 11 Department of Pharmacy, Mayo Clinic, Phoenix, Arizona; 12 Department of Cardiovascular Medicine, Mayo Clinic, Rochester, Minnesota; 13 Center for Digital Health, Mayo Clinic, Rochester, Minnesota; 14 Department of Neurosurgery, Mayo Clinic, Rochester, Minnesota; 15 Department of Emergency Medicine, Mayo Clinic, Jacksonville, Florida

## Abstract

**Importance:**

Direct oral anticoagulant (DOAC)–associated intracranial hemorrhage (ICH) has high morbidity and mortality. The safety and outcome data of DOAC reversal agents in ICH are limited.

**Objective:**

To evaluate the safety and outcomes of DOAC reversal agents among patients with ICH.

**Data Sources:**

PubMed, MEDLINE, The Cochrane Library, Embase, EBSCO, Web of Science, and CINAHL databases were searched from inception through April 29, 2022.

**Study Selection:**

The eligibility criteria were (1) adult patients (age ≥18 years) with ICH receiving treatment with a DOAC, (2) reversal of DOAC, and (3) reported safety and anticoagulation reversal outcomes. All nonhuman studies and case reports, studies evaluating patients with ischemic stroke requiring anticoagulation reversal or different dosing regimens of DOAC reversal agents, and mixed study groups with DOAC and warfarin were excluded.

**Data Extraction and Synthesis:**

Preferred Reporting Items for Systematic Reviews and Meta-analyses guidelines were used for abstracting data and assessing data quality and validity. Two reviewers independently selected the studies and abstracted data. Data were pooled using the random-effects model.

**Main Outcomes and Measures:**

The primary outcome was proportion with anticoagulation reversed. The primary safety end points were all-cause mortality and thromboembolic events after the reversal agent.

**Results:**

A total of 36 studies met criteria for inclusion, with a total of 1832 patients (967 receiving 4-factor prothrombin complex concentrate [4F-PCC]; 525, andexanet alfa [AA]; 340, idarucizumab). The mean age was 76 (range, 68-83) years, and 57% were men. For 4F-PCC, anticoagulation reversal was 77% (95% CI, 72%-82%; *I*^2^ = 55%); all-cause mortality, 26% (95% CI, 20%-32%; *I*^2^ = 68%), and thromboembolic events, 8% (95% CI, 5%-12%; *I*^2^ = 41%). For AA, anticoagulation reversal was 75% (95% CI, 67%-81%; *I*^2^ = 48%); all-cause mortality, 24% (95% CI, 16%-34%; *I*^2^ = 73%), and thromboembolic events, 14% (95% CI, 10%-19%; *I*^2^ = 16%). Idarucizumab for reversal of dabigatran had an anticoagulation reversal rate of 82% (95% CI, 55%-95%; *I*^2^ = 41%), all-cause mortality, 11% (95% CI, 8%-15%, *I*^2^ = 0%), and thromboembolic events, 5% (95% CI, 3%-8%; *I*^2^ = 0%). A direct retrospective comparison of 4F-PCC and AA showed no differences in anticoagulation reversal, proportional mortality, or thromboembolic events.

**Conclusions and Relevance:**

In the absence of randomized clinical comparison trials, the overall anticoagulation reversal, mortality, and thromboembolic event rates in this systematic review and meta-analysis appeared similar among available DOAC reversal agents for managing ICH. Cost, institutional formulary status, and availability may restrict reversal agent choice, particularly in small community hospitals.

## Key Points

**Question:**

What outcomes are associated with direct oral anticoagulant (DOAC) reversal agents in intracranial hemorrhage (ICH)?

**Findings:**

In a meta-analysis of 32 studies including 1832 patients with ICH, 4-factor prothrombin complex concentrate (4F-PCC), andexanet alfa (AA), and idarucizumab were associated with a successful anticoagulation reversal in 77%, 75%, and 82% of patients, respectively; all-cause mortality rates were 26%, 24%, and 11%, respectively; and thromboembolic event rates were 8%, 14%, and 5%, respectively. A direct retrospective comparison of 4F-PCC with AA showed no differences in successful anticoagulation reversal, all-cause mortality, or thromboembolic events.

**Meaning:**

In this study, factor Xa inhibitor reversal agents for ICH had similar safety profiles and outcomes, but the lack of head-to-head comparison warrants cautious interpretation.

## Teaser

This systematic review and meta-analysis evaluates the safety and outcomes of direct oral anticoagulation (DOAC) reversal agents among patients with intracranial hemorrhage (ICH).

## Introduction

The US Food and Drug Administration (FDA) has approved the direct thrombin inhibitor (DTI) dabigatran and factor Xa inhibitors rivaroxaban, edoxaban, and apixaban for nonvalvular atrial fibrillation (NVAF) to prevent stroke and systemic embolism and for prophylaxis of venous thromboembolism (VTE) for coronary artery disease (CAD) or peripheral artery disease to reduce the risk of major cardiovascular events.^1,2^ Over the last decade, direct oral anticoagulants (DOACs) have become preferred over warfarin.^3^ At the time of their approval, there were no specific antidotes to reverse DOAC-related bleeding, including intracranial hemorrhage (ICH).^4,5,6^ Annually, 0.1% to 0.2% of patients receiving DOACs experience ICH. This group’s risk factors for morbidity and mortality include older age, higher hematoma volumes, and hematoma expansion.^7,8,9^

Initially, nonspecific DOAC reversal agents such as fresh frozen plasma (FFP), activated prothrombin complex concentrate (A-PCC), and 4-factor PCC (4F-PCC) showed promising safety and anticoagulation reversal in DOAC-related bleeding.^10,11^ In 2015, the FDA approved the use of idarucizumab to reverse the effects of dabigatran,^12^ which marked the introduction of the first specific reversal agent for DOACs. In 2018, andexanet alfa was approved to reverse factor Xa inhibitors (FXaI).^13^ While the safety data from these landmark trials were promising, head-to-head trials have not compared the safety and outcomes of idarucizumab or andexanet alfa (AA) with traditional nonspecific reversal agents (FFP, 4F-PCC, or A-PCC). Such comparisons are limited to data generated from case series and cohort studies. This systematic review summarizes the safety and anticoagulation reversal success of non-specific and targeted DOAC reversal agents in patients with DOAC-related ICH.

## Methods

This systematic review was performed according to Cochrane Collaboration guidance. We followed the Preferred Reporting Items for Systematic Reviews and Meta-analyses (PRISMA) statement (eAppendix 1 in the Supplement).^14^

### Search Strategy

We searched PubMed, MEDLINE, The Cochrane Library, Embase, EBSCO, Web of Science, and CINAHL databases from the inception through April 29, 2022. All clinical studies evaluating the use of DOAC reversal agents in patients with ICH were included. The search strategy appears in eAppendix 2 in the Supplement. A strict prespecified protocol was followed to identify and systematically assess studies with the inclusion and exclusion criteria described in the next section.

### Eligibility Criteria

Two reviewers (Rahul C. and A.S.) independently selected and abstracted data from eligible studies on study design, patient demographic characteristics, anticoagulation reversal, mortality, and thromboembolic events. Discrepancies were resolved by discussion and consensus. The results were reviewed by senior investigators (A.N.A. and R.D.M.). The eligibility criteria were (1) adult patients (age ≥18 years) with ICH being treated with a DOAC, (2) reversal of DOAC, and (3) thromboembolic, mortality, or anticoagulation reversal outcomes. All nonhuman studies and case reports, studies with patients with ischemic stroke requiring anticoagulation reversal, studies evaluating different dosing regimens of reversal agents, and mixed study groups with DOAC and warfarin were excluded. Abstracts, conference presentations, editorials, reviews, expert opinions, and literature published in languages other than English were excluded.

### Outcome Definitions

The primary outcome was the successful reversal of anticoagulation. The primary safety end points were all-cause mortality and thromboembolic events after administration of the DOAC reversal agent.

### Risk-of-Bias Appraisal and Certainty of the Evidence

Qualitative bias evaluation was performed using the following key parameters for each study: (1) clear definition of the study population; (2) clear definition of outcomes and outcome assessment; (3) independent assessment of outcome parameters, and (4) identification of important confounders and prognostic factors. In the absence of randomized clinical trials, the risk of bias for all included cohort studies was assessed using the Newcastle-Ottawa Scale.^15^

Publication bias was estimated visually by funnel plots.^16,17^ If any bias was observed, further bias quantification was measured using the Begg-Mazumdar test,^18^ Egger test,^16^ and Duval-Tweedie trim-and-fill method.^19^ Sensitivity analyses were performed to assess the contribution of each study to the pooled estimate by excluding studies one at a time.

### Statistical Analysis

We anticipated considerable between-study heterogeneity, so a random-effects model using the generalized linear mixed models was used to pool effect sizes.^20^ The restricted maximum likelihood estimator^21^ was used to calculate the heterogeneity variance τ^2^. Knapp-Hartung adjustments^22^ were used to calculate the confidence interval around the pooled effect estimate. Relative risk (RR) was computed for the subanalysis comparing 4F-PCC with AA for the primary and safety end points. We evaluated the heterogeneity of effects using the Higgins *I*^2^ statistic with mild, moderate, and significant heterogeneity defined as 25%, 50%, and 75%, respectively.^23^ Meta-analysis was performed using R version 3.5.3 (R Foundation for Statistical Computing) and RStudio version 1.2.5003 (RStudio).

## Results

Thirty-six studies were included in the meta-analysis (eAppendix 1 in the Supplement). Twenty-two studies with 967 participants evaluated 4F-PCC for reversal^8,24,25,26,27,28,29,30,31,32,33,34,35,36,37,38,39,40,41,42,43,44,45^; 17 studies with 525 participants evaluated AA^13,35,36,37,39,40,41,42,44,46,47,48,49,50,51,52^; and 5 studies with 340 participants evaluated idarucizumab,^12,53,54,55,56^ consisting of a total of 1832 patients with ICH while receiving treatment with a DOAC. Eight studies had data on more than 1 agent.^35,36,37,39,40,41,42,44^ The characteristics of the studies are in the Table and eAppendix 3 in the Supplement.

**Table.** Type of ICH and Clinical Outcomes Among Included Studies

Source	Type of ICH, No./total No. (%)^a^				Outcomes, No./total No. (%)		
	SAH	SDH	Others (IPH/IVH/ICerH)	Traumatic ICH	Anticoagulation reversal^b^	Mortality	TE events
**4-Factor PCC**							
Grandhi et al,^24^ 2015	4/18 (22)	4/18 (22)	12/18 (67)	8/18 (44)	17/18 (94)	6/18 (33)	1/18 (6)
Majeed et al,^25^ 2017	NR	NR	NR	NR	43/59 (73)	13/59 (22)	3/59 (5)
Gerner et al,^8^ 2018	NR	NR	NR	NR	61/94 (65)	NR	NR
Schulman et al,^26^ 2017	NR	NR	NR	NR	30/36 (83)	8/36 (22)	NR
Tao et al,^27^ 2018	NR	NR	NR	NR	NR	NR	1/16 (6)
Sheikh-Taha,^28^ 2019	NR	NR	NR	NR	13/21 (62)	6/21 (29)	1/21 (5)
Smith et al,^29^ 2019	4/18 (22)	7/18 (39)	7/18 (39)	13/18 (72)	16/18 (89)	NR	0/18
Berger et al,^30^ 2020	7/22 (32)	5/22 (23)	7/22 (32)	NR	18/19 (95)	4/22 (18)	2/22 (9)
Zheng and Tormey,^31^ 2020	NR	NR	NR	NR	8/9 (89)	4/13 (31)	NR
Korobey et al,^32^ 2021	5/59 (8)	20/59 (34)	34/59 (58)	31/59 (53)	52/59 (88)	6/59 (10)	7/59 (12)
Castillo et al,^33^ 2021	10/67 (15)	25/67 (37)	30/67 (45)	44/67 (66)	59/67 (88)	5/67 (7)	0/67
Lipari et al,^34^ 2020	13/85 (15)	25/85 (29)	47/85 (55)	NR	72/85 (85)	12/85 (14)	2/85 (2)
Barra et al,^35^ 2020	0/11	3/11 (27)	8/11 (73)	5/11 (45)	6/10 (60)	7/11 (64)	1/11 (9)
Coleman et al,^36^ 2021	NR	NR	NR	NR	NR	43/170 (25)	NR
Ammar et al,^37^ 2021	0/16	0/16	13/16 (81)	8/16 (50)	6/10 (60)	6/16 (38)	0/16
Smythe et al,^38^ 2021	4/34 (12)	13/34 (38)	17/34 (50)	NR	21/29 (72)	6/34 (18)	3/34 (9)
Stevens et al,^39^ 2021	NR	NR	NR	NR	7/10 (70)	4/10 (40)	NR
Pasciolla et al,^45^ 2022	8/44 (18)	14/44 (32)	19/44 (43)	NR	14/19 (74)	18/44 (41)	5/44 (11)
Pham et al,^40^ 2022	13/62 (21)	23/62 (37)	58/62 (94)	NR	46/58 (79)	13/62 (21)	6/62 (10)
Parsels et al,^41^ 2022	11/26 (42)	5/26 (19)	9/26 (35)	18/26 (69)	23/26 (88)	NR	3/26 (12)
Milioglou et al,^42^ 2022	1/22 (5)	4/22 (18)	17/22 (77)	NR	NR	10/22 (45)	0/22
Dev et al,^43^ 2022	NR	NR	NR	NR	14/20 (70)	NR	NR
Vestal et al,^44^ 2022	6/35 (17)	1/35 (3)	28/35 (80)	11/35 (31)	17/31 (55)	13/35 (37)	10/35 (29)
**Andexanet alfa**							
Connolly et al,^13^ 2019; Demchuk et al,^50^ 2021	43/171 (25)	58/171 (34)	104/171 (61)	72/171 (42)	135/168 (80)	34/227 (15)	21/227 (9)
Stevens et al,^46^ 2019	NR	NR	NR	NR	3/6 (50)	2/6 (33)	NR
Culbreth et al,^49^ 2019	4/14 (29)	5/14 (36)	9/14 (64)	NR	8/14 (57)	5/14 (36)	NR
Brown et al,^47^ 2020	1/13 (8)	2/13 (15)	10/13 (77)	NR	10/11 (91)	4/13 (31)	0/13
Barra et al,^35^ 2020	1/18 (6)	5/18 (28)	12/18 (67)	12/18 (67)	16/18 (89)	4/18 (22)	3/18 (17)
Coleman et al,^36^ 2021	NR	NR	NR	NR	NR	6/67 (9)	NR
Giovino et al,^48^ 2020	4/39 (10)	16/39 (41)	19/39 (49)	24/39 (62)	29/39 (74)	4/35 (11)	2/35 (6)
Ammar et al,^37^ 2021	2/28 (7)	1/28 (4)	25/28 (89)	8/28 (29)	15/28 (54)	11/28 (39)	2/28 (7)
Stevens et al,^39^ 2021	NR	NR	NR	NR	4/7 (57)	NR	NR
Sobolewski et al,^51^ 2021	1/7 (14)	1/7 (14)	5/7 (71)	NR	5/7 (71)	3/7 (43)	2/7 (29)
Benz et al,^52^ 2022	NR	NR	NR	7/38 (18)	31/38 (82)	NR	NR
Pham et al,^40^ 2022	12/47 (26)	14/47 (30)	46/47 (98)	NR	31/38 (82)	16/47 (34)	4/47 (9)
Parsels et al,^41^ 2022	9/26 (35)	5/26 (19)	12/26 (46)	16/26 (62)	24/26 (92)	NR	7/26 (27)
Milioglou et al,^42^ 2022	0/23	4/23 (17)	19/23 (83)	NR	NR	11/23 (48)	0/23
Vestal et al,^44^ 2022	2/21 (10)	2/21 (10)	17/21 (81)	5/21 (24)	11/17 (65)	3/21 (14)	3/21 (14)
**Idarucizumab**							
Pollack et al,^12^ 2017	26/98 (27)	39/98 (40)	53/98 (54)	NR	NR	9/98 (9)	5/98 (5)
Sheikh-Taha et al,^53^ 2019	NR	NR	NR	2/6 (33)	4/6 (67)	0/6	0/6
Singh et al,^54^ 2020	NR	NR	NR	NR	NR	13/112 (12)	1/112 (1)
Kermer et al,^55^ 2020	2/40 (5)	11/40 (28)	27/40 (68)	NR	24/27 (89)	6/40 (15)	NR
Yasaka et al,^56^ 2020	25/84 (30)	34/84 (40)	47/84 (56)	NR	NR	9/84 (11)	5/84 (6)

Abbreviations: ICerH, intracerebral hemorrhage; ICH, intracranial hemorrhage; IPH, intraparenchymal hemorrhage; IVH, intraventricular hemorrhage; NR, not reported; PCC, prothrombin complex concentrate; SAH, subarachnoid hemorrhage; SDH, subdural hematoma; TE, thromboembolic.

^a^ Numbers add up to more than 100% since concomitant bleeding was present in more than one compartment.

^b^ If computed tomography head stability was measured at different time frames, hemostatic efficacy was calculated using the values for 24-hour after reversal agent administration to reduce heterogeneity in outcomes.

### Baseline Characteristics

Overall, the mean age was 76 years (68-83 years), and 57% were men. Individual characteristics of patients with ICH were unavailable for most studies (eAppendix 3 in the Supplement).

### Risk-of-Bias Assessment and Publication Bias

No significant publication bias was observed in the primary and safety outcomes analyses using funnel plots (eAppendix 4 in the Supplement). The sensitivity analysis revealed that a single study did not drive the results (eAppendix 5 in the Supplement). We deemed all studies at a high risk of bias because of unadjusted analyses and variability of comorbidities and prognostic factors in study groups.

### Primary Anticoagulation Reversal Outcomes

**Figure 1.**
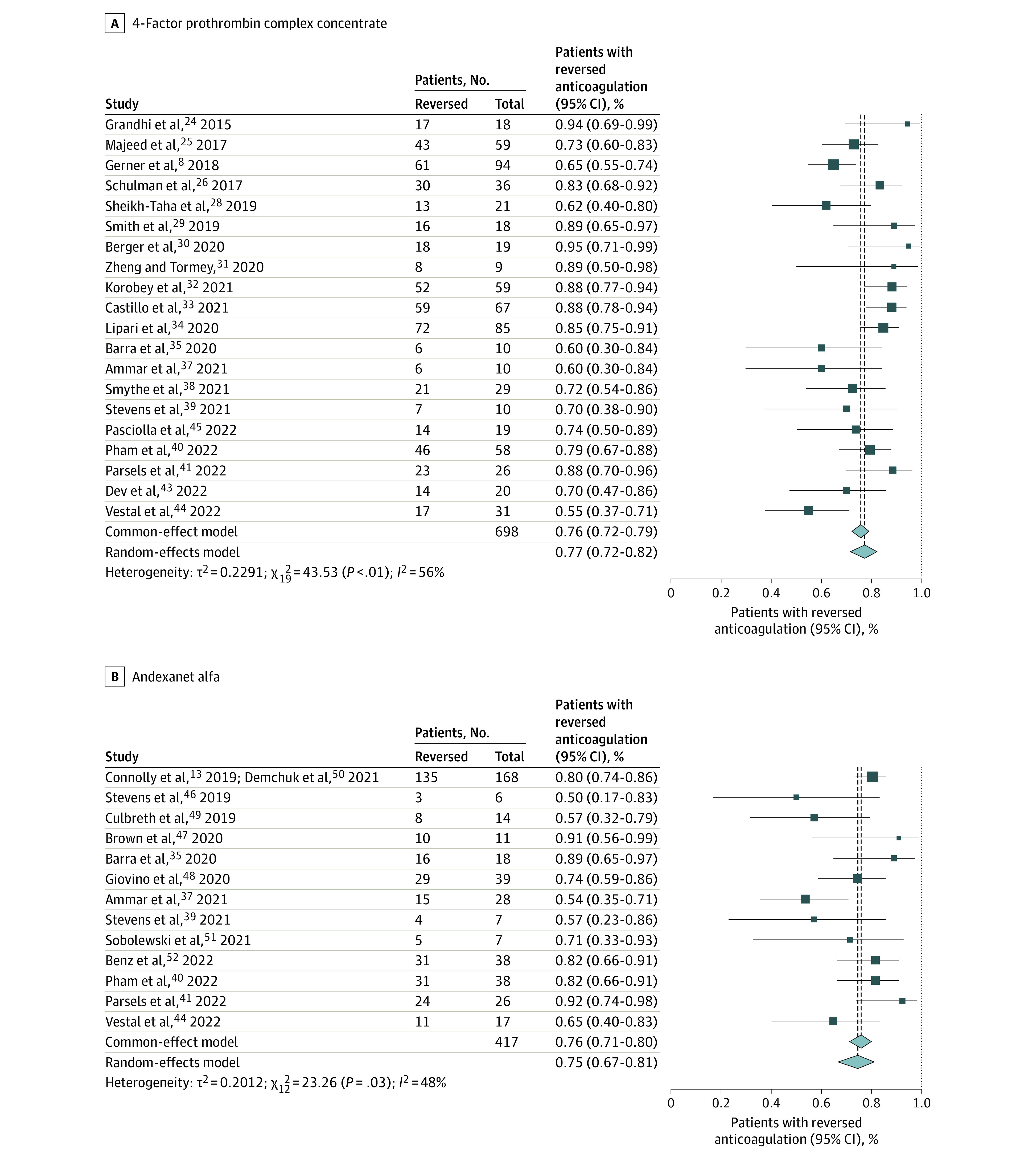
Primary Anticoagulation Reversal Outcomes for 4-Factor Prothrombin Complex Concentrate and Andexanet Alfa

The proportion with anticoagulation reversed for 4F-PCC in patients with ICH receiving a DOAC was 77% (95% CI, 72%-82%; *I*^2^ = 55%; 95% CI, 21%-83%). For AA, it was 75% (95% CI, 67%-81%; *I*^2^ = 48%; 95% CI, 0%-87%), and for idarucizumab for dabigatran reversal, it was 82% (95% CI, 55%-95%; *I*^2^ = 41%; 95% CI, 0% to >99%) (Figure 1).

### Primary Safety Outcomes

**Figure 2.**
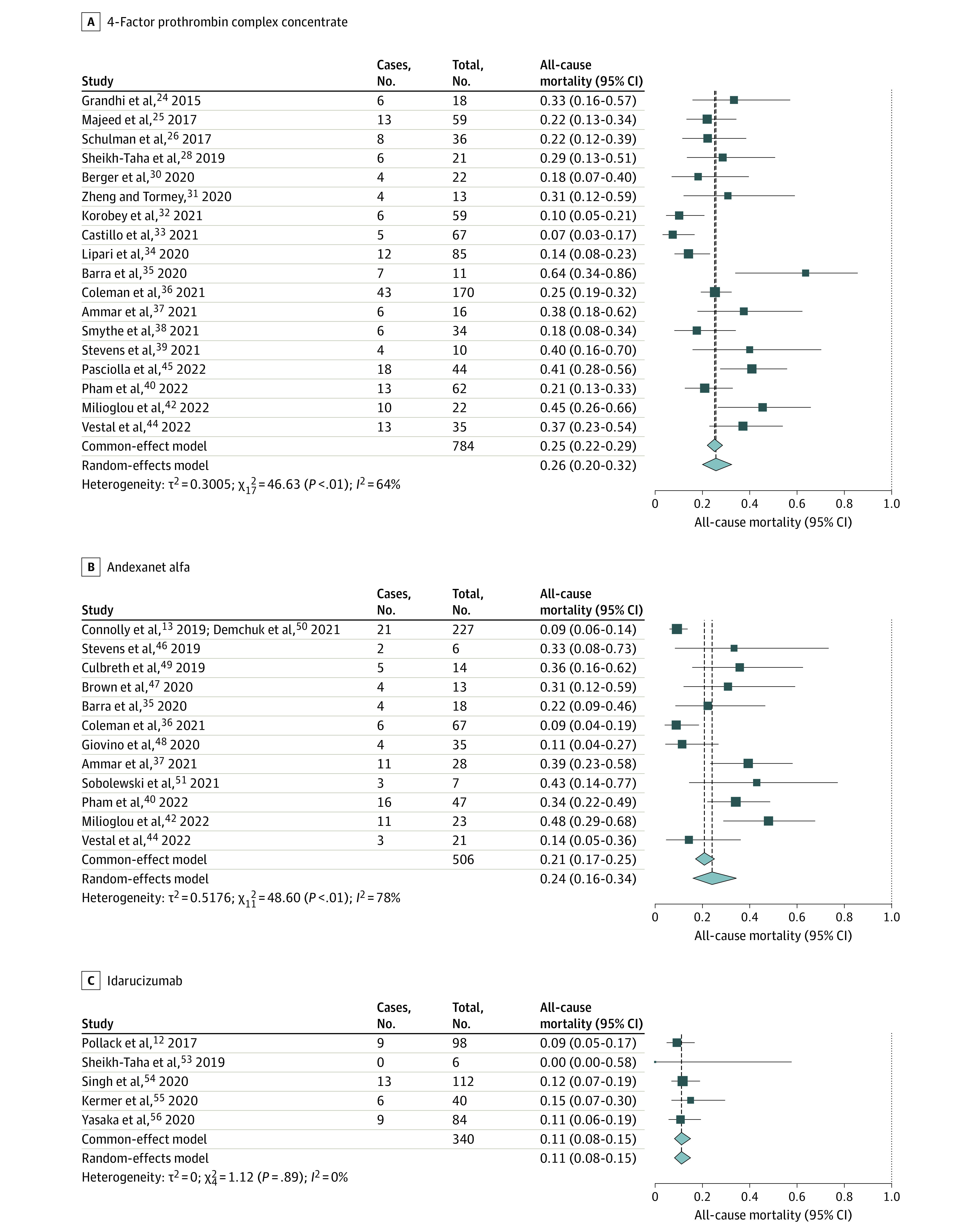
Mortality With 4-Factor Prothrombin Complex Concentrate, Andexanet Alfa, and Idarucizumab

All-cause mortality among patients with ICH who received 4F-PCC was 26% (95% CI, 20%-32%; *I*^2^ = 68%; 95% CI, 40%-88%). For AA, it was 24% (95% CI, 16%-34%; *I*^2^ = 73%; 95% CI, 42%-89%), and for idarucizumab for dabigatran reversal, it was 11% (95% CI, 8%-15%; *I*^2^ = 0%; 95% CI, 0%-62%) (Figure 2).

**Figure 3.**
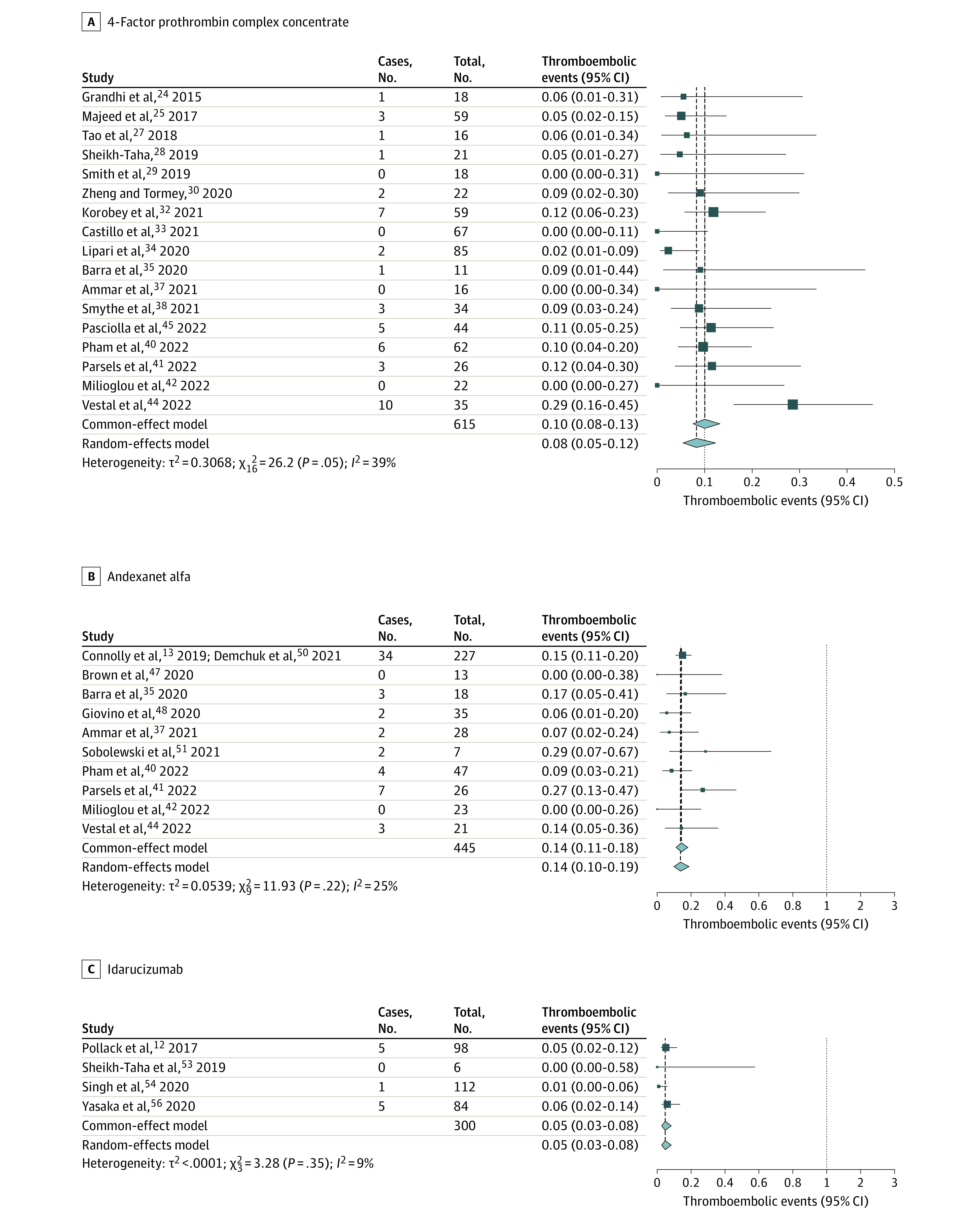
Thromboembolic Events With 4-Factor Prothrombin Complex Concentrate, Andexanet Alfa, and Idarucizumab

Thromboembolic events among patients receiving 4F-PCC was 8% (95% CI, 5%-12%, *I*^2^ = 41.3% 95% CI, 0%-71.8%). For AA, it was 14% (95% CI, 10%-19%; *I*^2^ = 16%; 95% CI, 0%-87.1%), and for idarucizumab, it was 5% (95% CI, 3%-8%, *I*^2^ = 0%; 95% CI, 0%-97%) (Figure 3).

### Subanalysis

**Figure 4.**
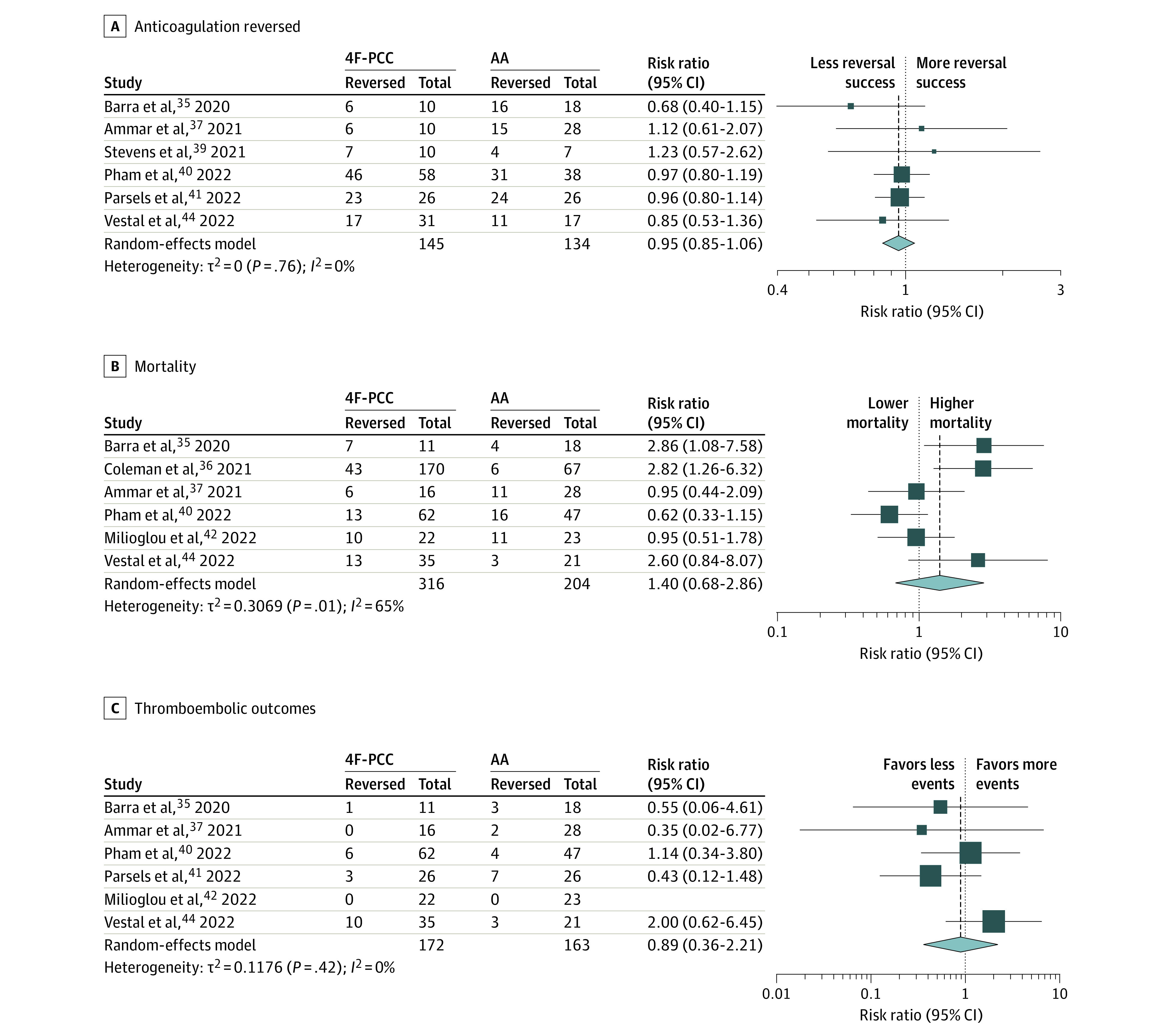
Comparison of 4-Factor Plasma Concentrate Complex (4F-PCC) vs Andexanet Alfa (AA) for Proportion of Anticoagulation Reversed, Mortality, and Thromboembolic Outcomes Among Patients Receiving Factor Xa Inhibitors With Intracranial Hemorrhage

Among the studies included for 4F-PCC safety and anticoagulation reversal outcome, 2 studies had a mixed population of FXaI and DTI^30,31^ with 17 patients. Patient-level data were unavailable to compare the primary and safety outcomes of 4F-PCC for reversing DTI. After the exclusion of these 2 studies, the data on the comparison of 4F-PCC and AA were derived from 8 retrospective studies.^35,36,37,39,40,41,42,44^ We found that 4F-PCC had a comparable proportion of successful anticoagulation reversal (RR, 0.95; 95% CI, 0.85-1.06; *I*^2^ = 0%; 95% CI, 0%-75%), proportional mortality (RR, 1.40; 95% CI, 0.68-2.86; *I*^2^ = 65%; 95% CI, 17%-86%), and thromboembolic event rate (RR, 0.89; 95% CI, 0.36-2.21, *I*^2^ = 0%; 95% CI, 0%-79%) when used for the reversal of FXaI in patients with ICH (Figure 4).

## Discussion

Among the studies included in our meta-analysis, the proportion of anticoagulation reversal of FXaI was comparable between 4F-PCC (77%; 95% CI, 72%-82%) and AA (75%; 95% CI, 67%-81%) for patients with ICH, including the subanalysis with direct comparison of 4F-PCC with AA (RR, 0.95; 95% CI, 0.85-1.06). While mortality rates were likewise similar for 4F-PCC (26%; 95% CI, 20%-32%) and AA (24%; 95% CI, 16%-34%), there may be a higher anticipated thromboembolic event rate following reversal with AA (14% vs 8%). However, the subanalysis comparing 4F-PCC with AA did not differ in all-cause mortality or thromboembolic rates. Idarucizumab, the reversal agent specific to dabigatran, achieved anticoagulation reversal in 82% (95% CI, 55%-95%) of patients with ICH, with low mortality (11%; 95% CI, 8%-15%) and thromboembolic complications (5%; 95% CI, 3%-8%).

ICH is an often-catastrophic complication of long-term anticoagulant therapy. The potential for ICH has long been limiting the widespread implementation of anticoagulation for patients with firm treatment indications, such as atrial fibrillation. In the absence of anticoagulant therapy, older adults who are most at risk of falling and suffering intracranial hemorrhage are also those at the most significant risk for thrombotic events. Fortunately, the risk of ICH is lower with DOAC than with warfarin. In a recent meta-analysis, dabigatran, apixaban, and rivaroxaban reduced ICH risk by 60%, 57%, and 41%, respectively, compared with warfarin.^57^ In NVAF trials, ICH rates were 0.10%/y to 0.44%/y for DOACs vs 0.38%/y to 0.90%/y for warfarin.^58,59^ In VTE trials, ICH rates were very low for patients receiving both DOAC (0%/y to 0.1%/y) and warfarin (0.2/y to 0.4%/y).^60,61^

Nevertheless, the consequences of ICH while taking a DOAC are considerable. In the preantidote era, 30-day mortality rates of 35% to 48% were noted.^62,63^ Despite the availability of proven reversal strategies for warfarin, these mortality rates were similar for DOACs and warfarin (4F-PCC, vitamin K, FFP).

### Four-Factor Prothrombin Complex Concentrates

Compared with FFP, 4F-PCCs contain approximately 25 times more vitamin K–dependent clotting factors (including factors II, VII, IX, and Xa). Rapid administration time, low volume infusion, leukocyte-free product, minimal risk for transfusion-related lung injury, and safety in heart failure all made 4F-PCCs favorable over FFP, and 4F-PCC was used for FXaI-related bleeding reversal before specific DOAC reversal agents were approved.

The RETRACE II study investigators^8^ evaluated the use of 4F-PCC in 146 patients with DOAC-related ICH across 19 tertiary care centers in Germany in a retrospective cohort design. Of these, 131 patients were receiving FXaI (rivaroxaban, apixaban), 15 were receiving dabigatran, and subsequently, 103 patients received 4F-PCC. Imaging evidence of hematoma enlargement (4F-PCC vs no 4F-PCC) was 33.3% vs 31.0% in rivaroxaban group, 46.2% vs 50.0% in apixaban group, and 22.2% vs 16.7% in the dabigatran group (*P* > .05 for all). The mortality rate was 19.9% at discharge and 29.5% at 3-month follow-up, with no significant differences between patients with and without PCC treatment. In another study, Coleman et al^36^ included 170 patients with DOAC-related ICH and reported a mortality rate of 25.3% in the 4F-PCC treatment cohort in 45 US hospitals.^36^

Several other smaller studies have shown the use of 4F-PCCs as a viable reversal option for DOAC-related ICH for anticoagulation reversal (reversal rates, 54.5%-94.7%) and safety (mortality rates, 4.7%-63.6%; thrombotic event rate, 0%-17.2%).^8,24,25,26,27,28,29,30,31,32,33,34,35,36,37,38,39,40,41,42,43,44,45^ Albeit with significant heterogeneity, our meta-analysis found a combined proportion of anticoagulation reversed of 77%, a mortality rate of 26%, and a thromboembolic event rate of 8% (Figures 1-3).

### Andexanet Alfa

AA received FDA approval in 2018. ANNEXA-4 was a multicentered, prospective study^13^ that enrolled 171 patients with FXaI-associated ICH and reported an anticoagulation reversal rate of 80.7%, a mortality rate of 14%, and a thromboembolic complication rate of 10%. Since then, multiple small retrospective studies in patients with ICH^13,35,36,37,39,40,41,42,44,46,47,48,49,50,51,52^ have reported rates of anticoagulation reversal between 50% and 91%; mortality, between 9% and 39%; and thrombotic complications, between 0% and 31%. Our meta-analysis showed a combined proportion of anticoagulation reversal of 75%, a mortality rate of 24%, and thromboembolic event rate of 14% (Figures 1-3).

The lack of control group data in most studies limits our understanding of AA’s comparative effectiveness and safety profile relative to 4F-PCC. The subanalysis of 8 studies^35,36,37,39,40,41,42,44^ that compared 4F-PCC with AA for DOAC reversal in patients with ICH showed no differences in safety or proportion of anticoagulation reversed between the reversal agents (Figure 4).

### Idarucizumab

The FDA approved idarucizumab in 2015 to reverse the anticoagulant effects of oral dabigatran. RE-VERSE AD^12^ was a multicentered, prospective cohort study of idarucizumab use in 98 patients with dabigatran-related ICH. They reported 100% anticoagulation reversal, with a 16.4% mortality rate and a 6.1% thromboembolic event rate.^12^ The rates of thrombosis were lower than those reported in 4F-PCC studies. Singh et al^54^ performed another retrospective cohort study comparing idarucizumab with no idarucizumab in 112 patients with dabigatran-related ICH.^54^ Mortality was 11.6% (vs 2.8% in patients who did not receive idarucizumab), and the thromboembolic event rate was 0.9% in this study. Confounding by disease severity was a major limitation affecting the interpretation of the study (ie, less severe cases might not have received reversal therapy). They also showed that idarucizumab use was associated with a reduced length of stay (incidence rate ratio [IRR], 0.82; *P* = .03) and higher hospitalization costs (IRR, 1.36; *P* = .01). Other smaller studies have shown a proportional anticoagulation reversal rate of 89% to 100%, a mortality rate of 0% to 15%, and a rate of thrombotic complications of 0% to 6%.^55,56^ Our meta-analysis found a combined anticoagulation reversal rate of 82%, a mortality rate of 11%, and a thromboembolic event rate of 5%.

### Strategies for Reversal

The individualization of the reversal strategy for DOACs in patients with ICH should consider (1) confirmation of the presence and extent of anticoagulation with DOACs, (2) pragmatic selection of patients, and (3) availability of specific DOAC reversal agents at the treating facility. For all FXaIs, the anti–factor Xa activity level can reliably assess the degree of anticoagulation, qualitatively and quantitatively, provided that the instruments are calibrated for the specific agents.^64^ However, anti–factor Xa levels specific to a given DOAC are unlikely to be available or will not be performed in a time frame that will affect decision-making at the bedside in the emergency setting in most institutions. As such, a generic chromogenic anti–factor Xa activity can rule out a meaningful level of any FXaI. A normal thrombin time usually helps exclude supratherapeutic anti-FXaI levels.^65,66^ Additionally, the measures of viscoelastic hemostatic assays, such as rotational thromboelastometry and thromboelastography, need to be further explored in emergencies.^67,68,69,70^ When available in a timely fashion, these assays may be especially useful in determining the need for a specific reversal agent in patients who have received 4F-PCC from a transferring facility.

Screening tests, such as the prothrombin time and activated partial thromboplastin clotting time, have high variability and are insufficient to exclude the presence of dabigatran.^71^ More sensitive assays for monitoring dabigatran activity include thrombin time, diluted thrombin time, and ecarin thrombin time. A normal thrombin time virtually excludes dabigatran presence. Although no international reference exists for calibration, mass spectrometry methods are considered the criterion standard for measuring dabigatran. However, these analyses require specialized laboratories, which often preclude their utility in life-threatening emergencies.

Pragmatic patient selection for DOAC reversal should include both severity of ICH and the time window for reversal. The factors for patient selection include ICH severity, anticipated risk of hematoma expansion, immediate need for surgical decompression, time since the last dose of DOAC, and creatinine clearance. The time window should be individualized based on clinical presentation and the patient’s rate of deterioration.

Finally, the availability of specific DOAC reversal agents in the treating facility warrants review and consideration. In a community setting with limited availability of AA and/or idarucizumab, non-specific reversal agents (ie, 4F-PCC) may be the only option. Other scenario-specific approaches may include activated charcoal (to remove the unabsorbed drug if the last ingestion was recent) and hemodialysis (for dabigatran removal).

### Future Directions

An ongoing randomized clinical trial (ANNEXa-I; NCT03661528) compares AA with usual care (expected to include administration of 4F-PCC in most cases) in patients with acute ICH associated with FXaI. Enrolling since January 2019, this study is expected to complete in 2023 and enroll approximately 900 participants. The primary outcome includes hemostatic efficacy at 12 hours. The secondary outcome comprises a change in baseline anti–factor Xa activity. This trial will provide more robust evidence of the relative benefit of the 2 DOAC reversal strategies for ICH.

Other reversal agents that may hold promise include ciraparantag, a small molecule with preferential binding to heparins and DOACs. To date, ciraparantag has only been studied in healthy human participants. Data from patients with anticoagulant-associated ICH in randomized clinical trials are needed to inform its use as a viable reversal option.

Current data are only available for a heterogeneous cohort of patients with DOAC-related ICH, including bleeding in various intracranial compartments (intraparenchymal and subdural), differing mechanisms (traumatic and nontraumatic), and insufficient information to determine the risk of hematoma expansion (higher risk in patients with more severe ICH and particularly in those who present early after symptom onset). These factors should be specifically addressed in future research. Additionally, reversal agent effectiveness and safety profile for patients requiring emergent surgical decompression (craniotomy or ventriculostomy) should be examined.

### Limitations

Our study has several limitations. First, patient-level data were not available for analysis. Second, smaller cohorts of case series and retrospective studies were included. Third, no comorbidities-matched head-to-head comparative trials are available between different reversal agents. Fourth, although most studies used the International Society of Thrombosis and Hemostasis criteria for assessing the effectiveness of major bleeding management,^72^ some studies used prespecified adjudication criteria or did not provide details of the criteria used to define the stability of ICH on repeated imaging (eAppendix 6 in the Supplement). Additionally, most studies had follow-up imaging at either 12 or 24 hours. When available, we used the 24-hour repeated imaging results to reduce heterogeneity. Fifth, the differences in the pathophysiology of intraparenchymal hemorrhage subtypes may affect the hemostatic effectiveness of a reversal agent. Similarly, some studies included traumatic and nontraumatic ICH that might have affected the outcome. Nonetheless, to our knowledge, this is the first study combining all available evidence of DOAC reversal strategies in patients with ICH. Prior meta-analyses have focused on the overall bleeding outcomes and have not specifically addressed ICH.^73,74^

## Conclusions

In this study, we evaluated the safety and anticoagulation reversal success measures of the available DOAC reversal agents to improve the understanding of their risk-benefit profile. Among patients presenting with ICH, the decision to reverse DOAC-associated anticoagulation and choice of agent should be individualized. Among the reversal agents, idarucizumab and AA are specific for the reversal of dabigatran and FXaIs, respectively. On the other hand, 4F-PCC is a nonspecific reversal agent studied predominantly in the reversal of FXaI. Overall, the proportion of anticoagulation reversed, mortality, and thromboembolic event rates appear similar between 4F-PCC and AA for FXaI reversal. However, the lack of head-to-head comparison warrants cautious interpretation.

4F-PCC is more readily available in some community settings and cheaper.^75^ Randomized clinical trials directly comparing the effectiveness and safety of 4F-PCC with specific reversal agents, ie, idarucizumab or AA, are needed to determine the optimal reversal strategies for patients with DOAC-related ICH. Additional research is needed to optimize patient-centered best practices and improve outcomes for those requiring reversal therapy for ICH.

## Notes

Supplement. **eAppendix 1.** Process of Study Selection for Clinical Trials **eAppendix 2.** Search Strategy **eAppendix 3.** Baseline Characteristics of Included Studies **eAppendix 4.** Funnel Plots for Primary Outcome of Anticoagulation Reversal and Primary Safety Outcomes for 4F-PCC, Andexanet Alfa (AA), and Idarucizumab **eAppendix 5.** Sensitivity Analysis for Primary Outcome of Anticoagulation Reversal and Primary Safety Outcomes for 4F-PCC, Andexanet Alfa (AA), and Idarucizumab **eAppendix 6.** Definitions of Successful Anticoagulation Reversal in Included Studies Included in the Meta-analysis
