# Supplementary material for: Evaluation of Direct Oral Anticoagulant Reversal Agents in Intracranial Hemorrhage: A Systematic Review and Meta-analysis
Source: JAMA Netw Open. 2022 Nov 4;5(11):e2240145. doi: 10.1001/jamanetworkopen.2022.40145 (PMC9636520; doi:10.1001/jamanetworkopen.2022.40145)
Supplement: Supplement. — eAppendix 1. Process of Study Selection for Clinical Trials eAppendix 2. Search Strategy eAppendix 3. Baseline Characteristics of Included Studies eAppendix 4. Funnel Plots for Primary Outcome of Anticoagulation Reversal and Primary Safety Outcomes for 4F-PCC, Andexanet Alfa (AA), and Idarucizumab eAppendix 5. Sensitivity Analysis for Primary Outcome of Anticoagulation Reversal and Primary Safety Outcomes for 4F-PCC, Andexanet Alfa (AA), and Idarucizumab eAppendix 6. Definitions of Successful Anticoagulation Reversal in Included Studies Included in the Meta-analysis [file jamanetwopen-e2240145-s001.pdf]

Supplemental Online Content

Chaudhary R, Singh A, Chaudhary R, et al. Evaluation of direct oral anticoagulant reversal agents in intracranial hemorrhage: a systematic review and meta-analysis. *JAMA Netw Open.* 2022;5(11):e2240145. doi:10.1001/jamanetworkopen.2022.40145

eAppendix 1. Process of Study Selection for Clinical Trials

eAppendix 2. Search Strategy

eAppendix 3. Baseline Characteristics of Included Studies

eAppendix 4. Funnel Plots for Primary Outcome of Anticoagulation Reversal and Primary Safety Outcomes for 4F-PCC, Andexanet Alfa (AA), and Idarucizumab

eAppendix 5. Sensitivity Analysis for Primary Outcome of Anticoagulation Reversal and Primary Safety Outcomes for 4F-PCC, Andexanet Alfa (AA), and Idarucizumab

eAppendix 6. Definitions of Successful Anticoagulation Reversal in Included Studies Included in the Meta-analysis

This supplemental material has been provided by the authors to give readers additional information about their work.

© 2022 Chaudhary R et al. *JAMA Network Open.*

eAppendix 1. Process of Study Selection for Clinical Trials

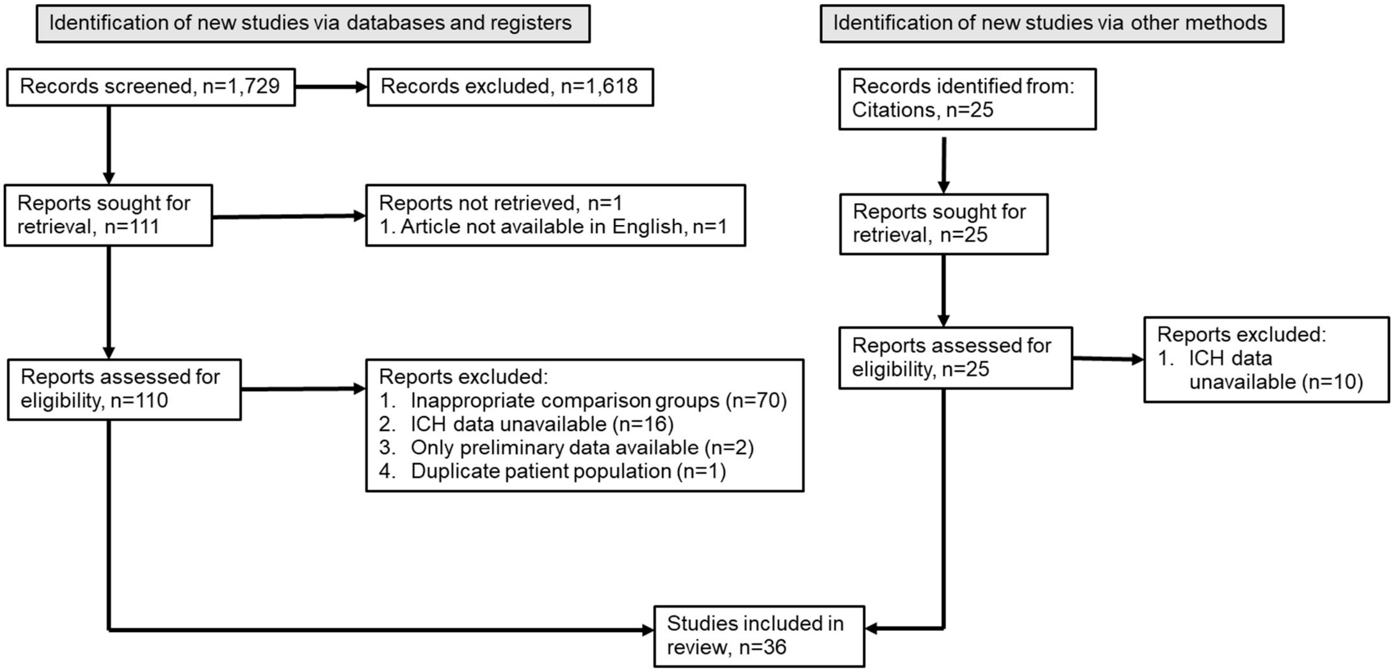

© 2022 Chaudhary R et al. *JAMA Network Open.*

eAppendix 2. Search Strategy

("DOAC"[All Fields] OR (("direct"[All Fields] OR "directed"[All Fields] OR "directing"[All Fields] OR "direction"[All Fields] OR "directional"[All Fields] OR "directions"[All Fields] OR "directivities"[All Fields] OR "directivity"[All Fields] OR "directs"[All Fields]) AND ("mouth"[MeSH Terms] OR "mouth"[All Fields] OR "oral"[All Fields]) AND ("anticoagulants"[Pharmacological Action] OR "anticoagulants"[MeSH Terms] OR "anticoagulants"[All Fields] OR "anticoagulant"[All Fields] OR "anticoagulate"[All Fields] OR "anticoagulated"[All Fields] OR "anticoagulating"[All Fields] OR "anticoagulation"[All Fields] OR "anticoagulations"[All Fields] OR "anticoagulative"[All Fields])) OR ("rivaroxaban"[MeSH Terms] OR "rivaroxaban"[All Fields]) OR ("apixaban"[Supplementary Concept] OR "apixaban"[All Fields] OR "apixaban s"[All Fields]) OR ("betrixaban"[Supplementary Concept] OR "betrixaban"[All Fields]) OR ("dabigatran"[MeSH Terms] OR "dabigatran"[All Fields] OR "dabigatran s"[All Fields]) OR (("novel"[All Fields] OR "novel s"[All Fields] OR "novels"[All Fields]) AND ("mouth"[MeSH Terms] OR "mouth"[All Fields] OR "oral"[All Fields]) AND ("anticoagulants"[Pharmacological Action] OR "anticoagulants"[MeSH Terms] OR "anticoagulants"[All Fields] OR "anticoagulant"[All Fields] OR "anticoagulate"[All Fields] OR "anticoagulated"[All Fields] OR "anticoagulating"[All Fields] OR "anticoagulation"[All Fields] OR "anticoagulations"[All Fields] OR "anticoagulative"[All Fields])) OR ("n 4 oleylcytosine arabinoside"[Supplementary Concept] OR "n 4 oleylcytosine arabinoside"[All Fields] OR "noac"[All Fields]) OR ("edoxaban"[Supplementary Concept] OR "edoxaban"[All Fields])) AND ((("DOAC"[All Fields] OR (("direct"[All Fields] OR "directed"[All Fields] OR "directing"[All Fields] OR "direction"[All Fields] OR "directional"[All Fields] OR

© 2022 Chaudhary R et al. *JAMA Network Open.*

"directions"[All Fields] OR "directivities"[All Fields] OR "directivity"[All Fields] OR "directs"[All Fields]) AND ("mouth"[MeSH Terms] OR "mouth"[All Fields] OR "oral"[All Fields]) AND ("anticoagulants"[Pharmacological Action] OR "anticoagulants"[MeSH Terms] OR "anticoagulants"[All Fields] OR "anticoagulant"[All Fields] OR "anticoagulate"[All Fields] OR "anticoagulated"[All Fields] OR "anticoagulating"[All Fields] OR "anticoagulation"[All Fields] OR "anticoagulations"[All Fields] OR "anticoagulative"[All Fields])) OR ("rivaroxaban"[MeSH Terms] OR "rivaroxaban"[All Fields]) OR ("apixaban"[Supplementary Concept] OR "apixaban"[All Fields] OR "apixaban s"[All Fields]) OR ("betrixaban"[Supplementary Concept] OR "betrixaban"[All Fields]) OR ("dabigatran"[MeSH Terms] OR "dabigatran"[All Fields] OR "dabigatran s"[All Fields]) OR (("novel"[All Fields] OR "novel s"[All Fields] OR "novels"[All Fields]) AND ("mouth"[MeSH Terms] OR "mouth"[All Fields] OR "oral"[All Fields]) AND ("anticoagulants"[Pharmacological Action] OR "anticoagulants"[MeSH Terms] OR "anticoagulants"[All Fields] OR "anticoagulant"[All Fields] OR "anticoagulate"[All Fields] OR "anticoagulated"[All Fields] OR "anticoagulating"[All Fields] OR "anticoagulation"[All Fields] OR "anticoagulations"[All Fields] OR "anticoagulative"[All Fields])) OR ("n 4 oleylcytosine arabinoside"[Supplementary Concept] OR "n 4 oleylcytosine arabinoside"[All Fields] OR "noac"[All Fields]) OR ("edoxaban"[Supplementary Concept] OR "edoxaban"[All Fields])) AND ("reversal"[All Fields] OR "reversals"[All Fields] OR "reverse"[All Fields] OR "reversed"[All Fields] OR "reversely"[All Fields] OR "reverses"[All Fields] OR "reversibilities"[All Fields] OR "reversibility"[All Fields] OR "reversible"[All Fields] OR "reversing"[All Fields] OR "reversion"[All Fields] OR "reversions"[All Fields])) OR ("prt064445"[Supplementary Concept] OR "prt064445"[All Fields] OR "andexanet alfa"[All

Fields]) OR ("idarucizumab"[Supplementary Concept] OR "idarucizumab"[All Fields]) OR ("factor ix"[MeSH Terms] OR ("factor"[All Fields] AND "ix"[All Fields]) OR "factor ix"[All Fields] OR ("prothrombin"[All Fields] AND "complex"[All Fields] AND "concentrate"[All Fields]) OR "prothrombin complex concentrate"[All Fields]))

Translations

1. direct: "direct"[All Fields] OR "directed"[All Fields] OR "directing"[All Fields] OR "direction"[All Fields] OR "directional"[All Fields] OR "directions"[All Fields] OR "directivities"[All Fields] OR "directivity"[All Fields] OR "directs"[All Fields]

2. oral: "mouth"[MeSH Terms] OR "mouth"[All Fields] OR "oral"[All Fields]

3. anticoagulants: "anticoagulants"[Pharmacological Action] OR "anticoagulants"[MeSH Terms] OR "anticoagulants"[All Fields] OR "anticoagulant"[All Fields] OR "anticoagulate"[All Fields] OR "anticoagulated"[All Fields] OR "anticoagulating"[All Fields] OR "anticoagulation"[All Fields] OR "anticoagulations"[All Fields] OR "anticoagulative"[All Fields]

4. rivaroxaban: "rivaroxaban"[MeSH Terms] OR "rivaroxaban"[All Fields] OR "rivaroxaban's"[All Fields]

1. apixaban: "apixaban"[Supplementary Concept] OR "apixaban"[All Fields] OR "apixaban's"[All Fields]

2. betrixaban: "betrixaban"[Supplementary Concept] OR "betrixaban"[All Fields]

3. dabigatran: "dabigatran"[MeSH Terms] OR "dabigatran"[All Fields] OR "dabigatran's"[All Fields]

4. novel: "novel"[All Fields] OR "novel's"[All Fields] OR "novels"[All Fields]

5. oral: "mouth"[MeSH Terms] OR "mouth"[All Fields] OR "oral"[All Fields]

1. anticoagulant: "anticoagulants"[Pharmacological Action] OR "anticoagulants"[MeSH Terms] OR "anticoagulants"[All Fields] OR "anticoagulant"[All Fields] OR "anticoagulate"[All Fields] OR "anticoagulated"[All Fields] OR "anticoagulating"[All Fields] OR "anticoagulation"[All Fields] OR "anticoagulations"[All Fields] OR "anticoagulative"[All Fields]

2. reversal: "reversal"[All Fields] OR "reversals"[All Fields] OR "reverse"[All Fields] OR "reversed"[All Fields] OR "reversely"[All Fields] OR "reverses"[All Fields] OR "reversibilities"[All Fields] OR "reversibility"[All Fields] OR "reversible"[All Fields] OR "reversing"[All Fields] OR "reversion"[All Fields] OR "reversions"[All Fields]

3. andexanet alfa: "PRT064445"[Supplementary Concept] OR "PRT064445"[All Fields] OR "andexanet alfa"[All Fields]

4. idarucizumab: "idarucizumab"[Supplementary Concept] OR "idarucizumab"[All Fields]

5. Prothrombin complex concentrate: "factor ix"[MeSH Terms] OR ("factor"[All Fields] AND "ix"[All Fields]) OR "factor ix"[All Fields] OR ("prothrombin"[All Fields] AND "complex"[All Fields] AND "concentrate"[All Fields]) OR "prothrombin complex concentrate"[All Fields]

© 2022 Chaudhary R et al. *JAMA Network Open.*

eAppendix 3. Baseline Characteristics of Included Studies

Name of Study	Study design	Total patients	Age (Mean)	Men (%)	Indication for Anticoagulation		
					Atrial Fibrillation (%)	Venous Thromboembolism (%)	Others (%)
**Andexanet Alfa**							
Connolly et al,13 2019	Prospective	171	-	-	-	-	-
Stevens et al,46 2019	Retrospective	6	69	-	-	-	-
Culbreth et al,49 2019	Prospective	14	82	-	71%	-	-
Brown et al,47 2020	Retrospective	13	75	46.2%	46.2%	53.8%	0%
Barra et al,35 2020	Retrospective	18	83	55.6%	94.4%	11.1%	0%
Coleman et al,36 2020	Retrospective	67	-	-	-	-	-
Giovino et al,48 2020	Retrospective	39	81	61.5%	94.9%	18%	2.6%
Ammar et al,37 2021	Retrospective	28	78	61%	75%	21%	4%
Stevens et al,39 2021	Retrospective	32	69	25%	34%	16%	50%
Demchuk et al,50 2021	Sub-analysis of ANNEXA-4	227	79	52%	85%	13%	2%
Sobolewski et al,51 2021	Retrospective	7	75	86%	-	-	-
Pham et al,40 2021	Retrospective	47	77	66%	83%	13%	4%
Parsels et al,41 2022	Retrospective	26	83	39%	77%	23%	0%
Milioglou et al,42 2022	Retrospective	23	76	57%	87%	13%	0%
Benz et al,52 2022	Prospective	36	-	-	-	-	-
Vestal et al,44 2022	Retrospective	21	73	71%	67%	33%	0%
**Idarucizumab**							
Pollack et al,12 2017	Prospective	98	-	-	-	-	-
Sheikh-Taha et al,52 2019	Retrospective	6	80	83.3%	100%	0%	0%
Singh et al,54 2020	Retrospective	112	76	57.1%	-	-	-
Kermer et al,55 2020	Retrospective	40	77	70%	100%	0%	0%
Yasaka et al,56 2020	Retrospective	84	-	-	-	-	-
**4F-PCC**							
Grandhi et al,24 2015	Retrospective	18	79.7	55.6%	88.9%	5.6%	1.7%

Majeed et al,25 2017	Prospective	59	75	-	-	-	-
Gerner et al,8 2018	Retrospective	146	77.4	52.7%	-	-	-
Schulman et al,26 2018	Prospective	36	76.9	-	-	-	-
Tao et al,27 2018	Retrospective	16	74	-	-	-	-
Sheikh-Taha et al,28 2018	Retrospective	21	73.8	-	-	-	-
Smith et al,29 2019	Retrospective	18	74	-	-	-	-
Berger et al,30 2020	Retrospective	22	79.5	50%	77.2%	27.3%	18.2%
Zheng et al,31 2020	Retrospective	13	68.3	-	-	-	-
Korobey et al,32 2020	Retrospective	59	78.5	60%	83.1%	27.1%	0%
Castillo et al,33 2020	Retrospective	67	77.3	56.7%	77.6%	10.5%	9%
Lipari et al,34 2020	Retrospective	85	77	-	-	-	-
Barra et al,35 2020	Retrospective	11	71	81.8%	72.7%	27.3%	0%
Coleman et al,36 2020	Retrospective	170	70.1	-	-	-	-
Ammar et al,37 2021	Retrospective	16	80	69%	81%	19%	0%
Smythe et al,38 2021	Retrospective	29	-	-	-	-	
Stevens et al,39 2021	Retrospective	32	-	34.4%	43.8%	6.3%	50%
Pasciolla et al,45 2021	Retrospective	44	78	64%	71%	25%	
Pham et al,40 2021	Retrospective	62	81	51.6%	77.4%	16.1%	6.5%
Parsels et al,41 2022	Retrospective	26	77	57.7%	76.9%	23.1%	0%
Milioglou et al,42 2022	Retrospective	22	77	63.6%	90.9%	9.1%	0%
Dev et al,43 2022	Retrospective	20	-	-	-	-	-
Vestal et al,44 2022	Retrospective	35	74.5	40%	71.4%	25.7%	2.9%

© 2022 Chaudhary R et al. *JAMA Network Open.*

eAppendix 4. Funnel Plots for Primary Outcome of Anticoagulation Reversal and Primary Safety Outcomes for 4F-PCC, Andexanet Alfa (AA), and Idarucizumab

1a. 4-FPCC anticoagulation reversal

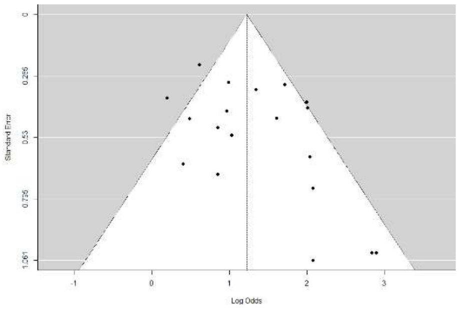

1b. 4-FPCC all-cause mortality

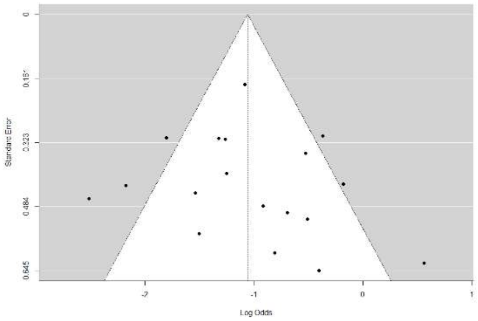

1c. 4F-PCC thromboembolic events

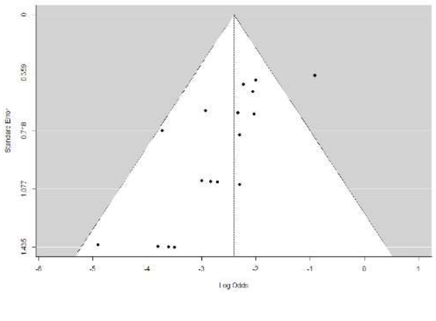

2a. AA anticoagulation reversal

© 2022 Chaudhary R et al. *JAMA Network Open.*

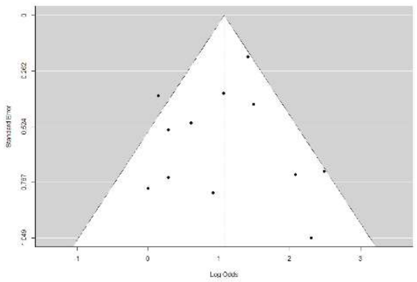

2b. AA all-cause mortality

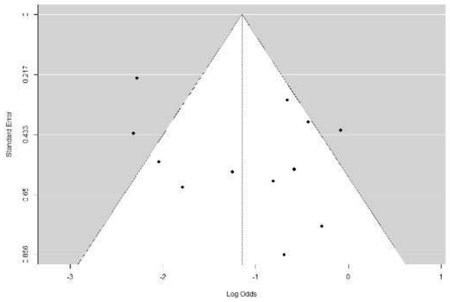

2c. AA thromboembolic events

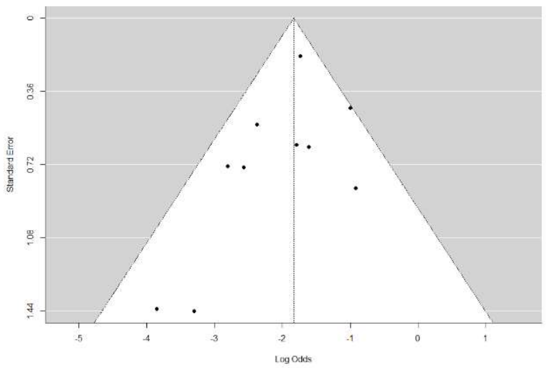

3a. Idarucizumab anticoagulation reversal

© 2022 Chaudhary R et al. *JAMA Network Open.*

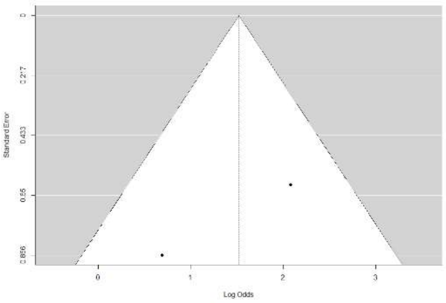

3b. Idarucizumab all-cause mortality

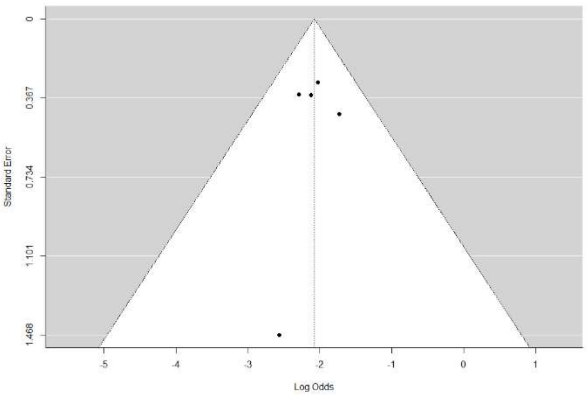

3c. Idarucizumab thromboembolic events

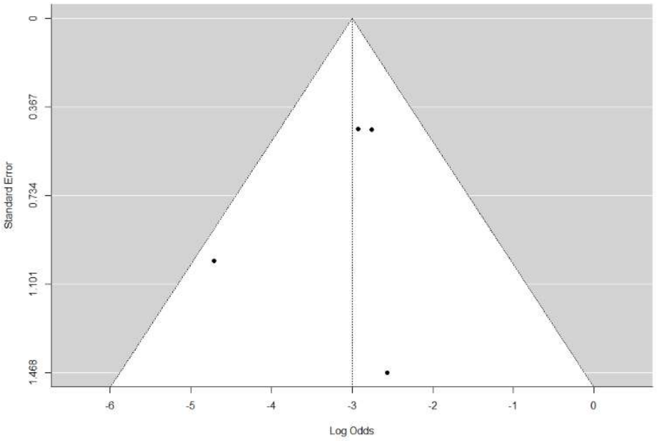

4a. 4F-PCC vs. andexanet alfa anticoagulation reversal

© 2022 Chaudhary R et al. *JAMA Network Open.*

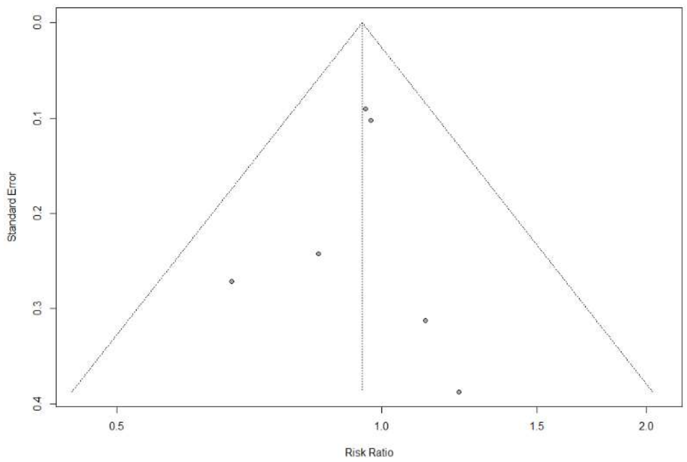

4b. 4F-PCC vs. andexanet alfa all-cause mortality

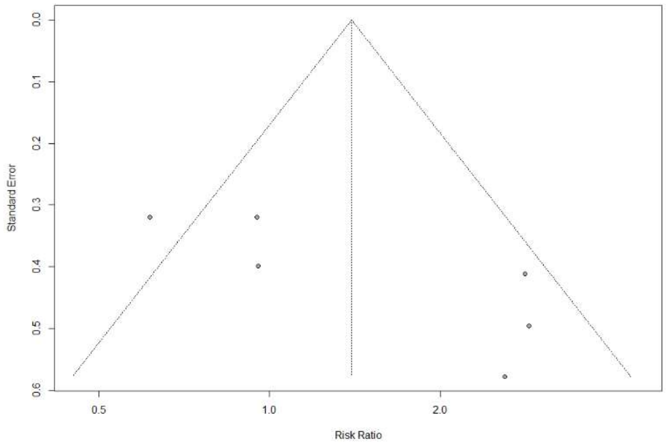

4c. 4F-PCC vs. andexanet alfa thromboembolic outcomes

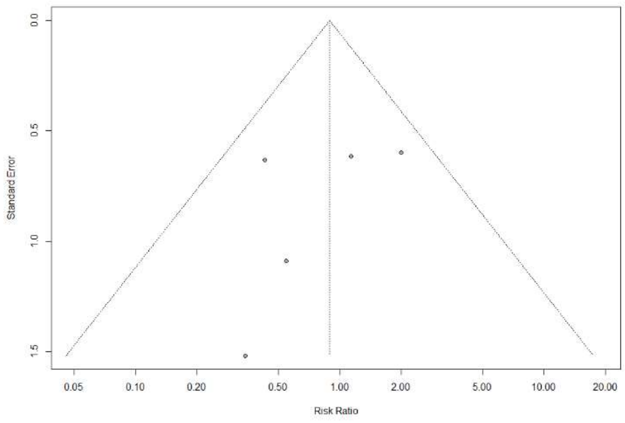

© 2022 Chaudhary R et al. *JAMA Network Open.*

eAppendix 5. Sensitivity Analysis for Primary Outcome of Anticoagulation Reversal and Primary Safety Outcomes for 4F-PCC, Andexanet Alfa (AA), and Idarucizumab

1a. 4-FPCC anticoagulation reversal

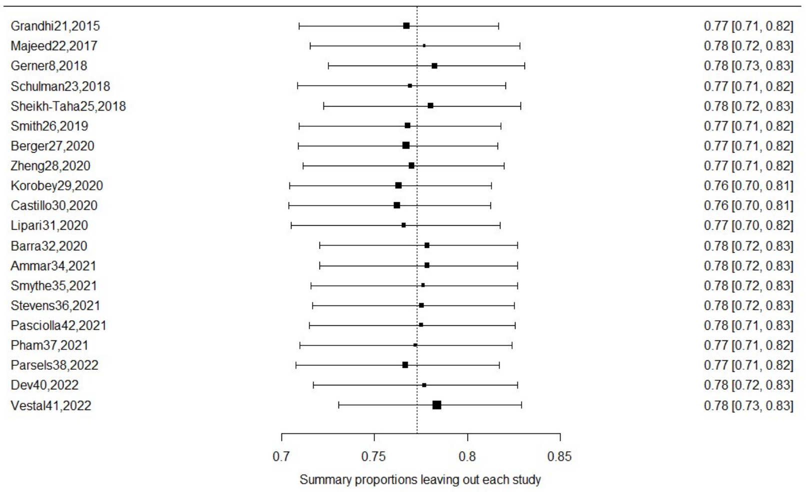

1b. 4-FPCC all-cause mortality

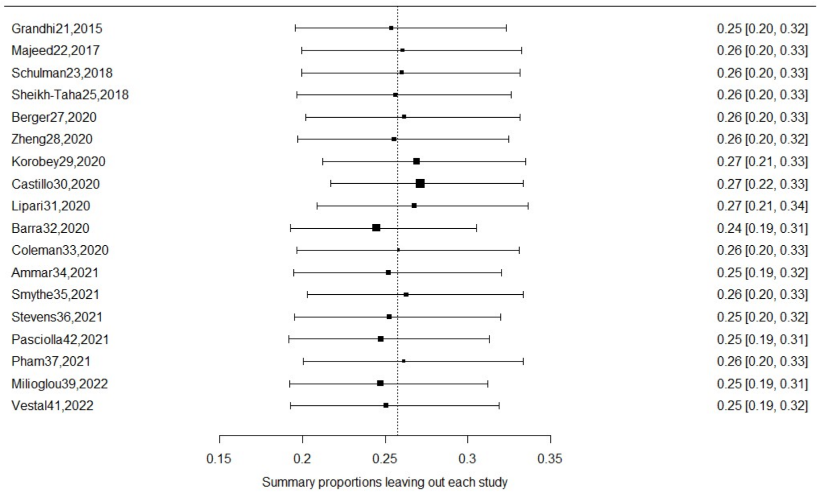

1c. 4F-PCC thromboembolic events

© 2022 Chaudhary R et al. *JAMA Network Open.*

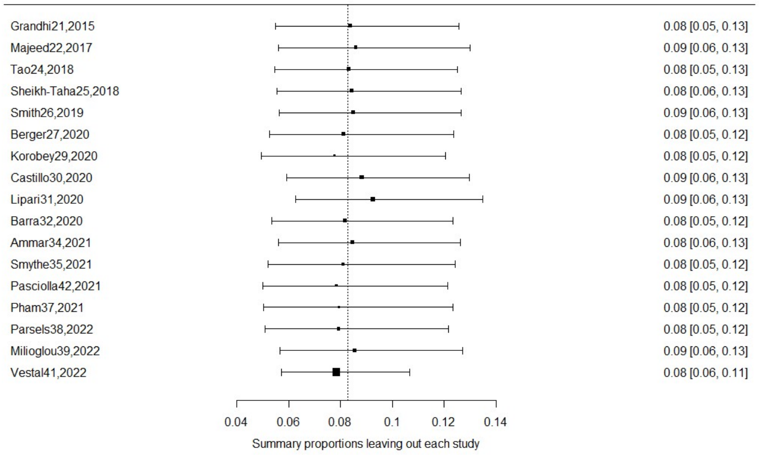

2a. AA anticoagulation reversal

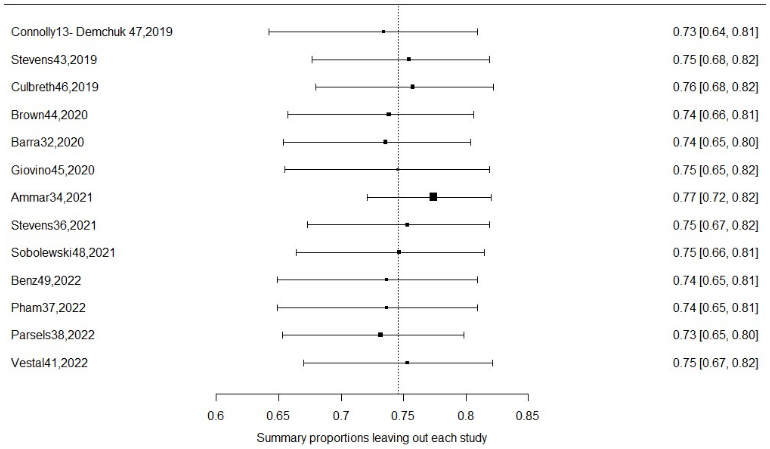

2b. AA all-cause mortality

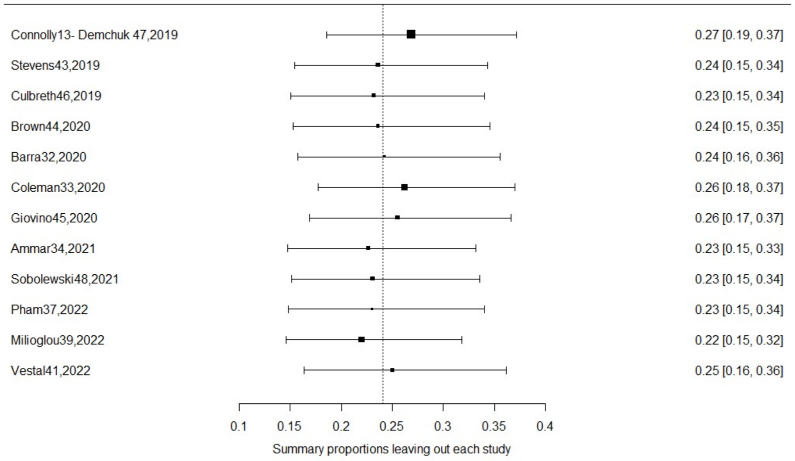

© 2022 Chaudhary R et al. *JAMA Network Open.*

2c. AA thromboembolic events

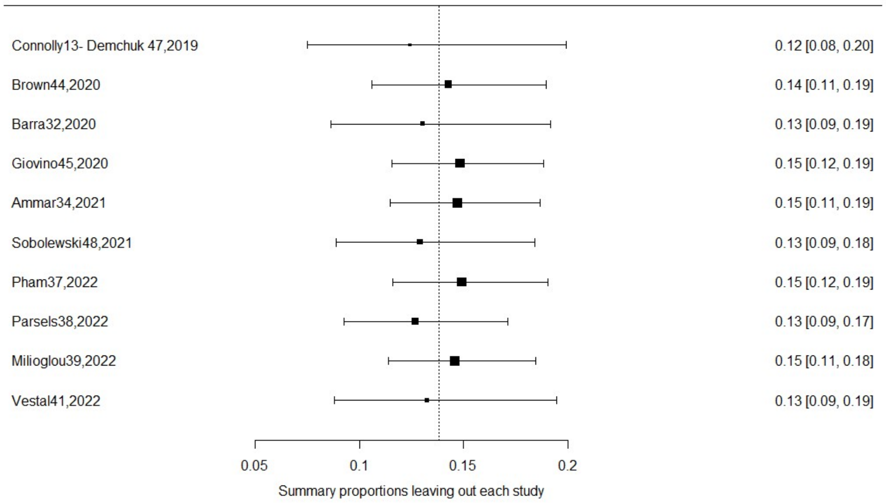

3a. Idarucizumab anticoagulation reversal

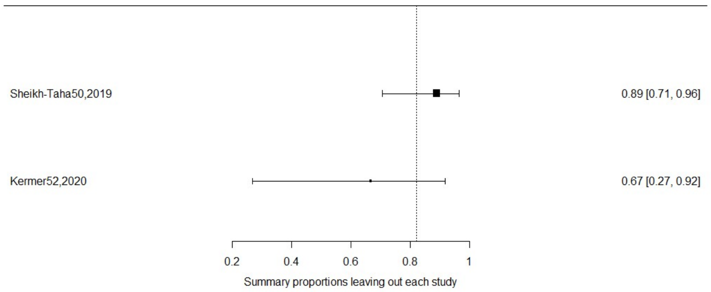

3b. Idarucizumab all-cause mortality

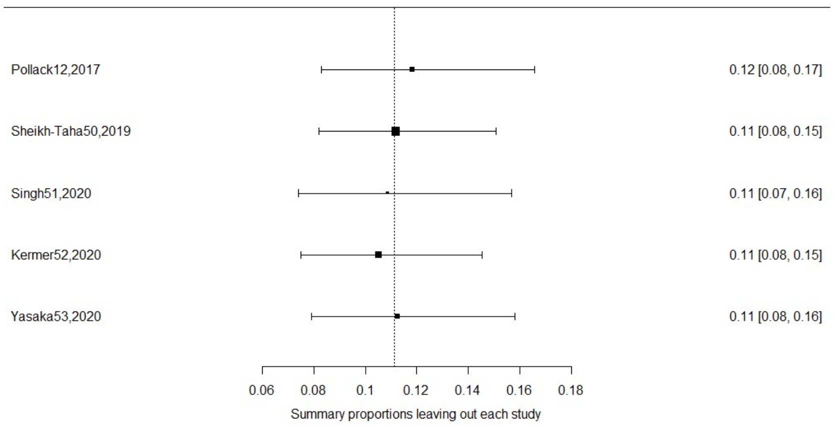

3c. Idarucizumab thromboembolic events

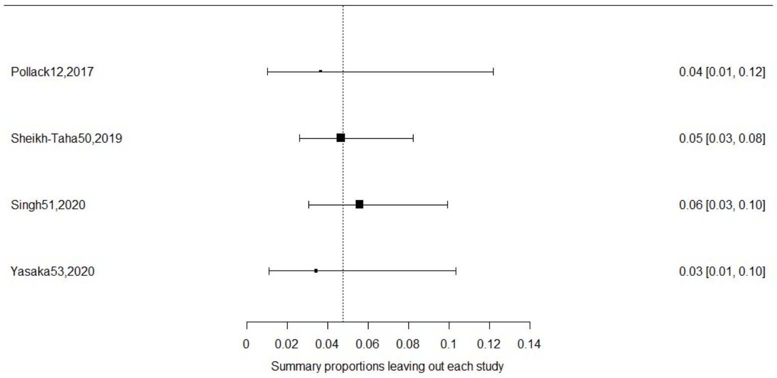

© 2022 Chaudhary R et al. *JAMA Network Open.*

eAppendix 6. Definitions of Successful Anticoagulation Reversal in Included Studies Included in the Meta-analysis

Study name	Hemostasis definition on brain imaging
**Andexanet alfa**	
Connolly et al,13 2019 **(ANNEXA-4)** + Demchuk et al,50 2021	**Intracerebral hematoma**:<br>Excellent: ≤20% increase in hematoma volume compared to baseline on a repeat CT or MRI scan performed at both the 1- and **12-hour** post infusion time points<br>Good: >20% but ≤35% increase in hematoma volume compared to baseline on a repeat CT or MRI scan at +12-hour time point<br><br>**Subarachnoid hemorrhage:**<br>Excellent: ≤20% increase in maximum thickness using the most dense area on the follow-up vs baseline at both the 1- and 12-hour post infusion time points<br>Good: >20% but <35% increase in maximum thickness using the most dense area on the follow-up at +12h vs baseline<br><br>**Subdural hematoma:**<br>Excellent: ≤20% increase in maximum thickness at both the 1- and 12-hour post infusion assessments compared to baseline<br>Good: >20% but < 35% increase in maximum thickness at +12h compared to baseline
Stevens et al,46 2019	**Intracerebral hematoma**:<br>Excellent: ≤20% increase in hematoma volume compared to baseline on a repeat CT or MRI scan performed at both the 1- and **12-hour** post infusion time points<br>Good: >20% but ≤35% increase in hematoma volume compared to baseline on a repeat CT or MRI scan at +12-hour time point<br><br>**Subarachnoid hemorrhage:**<br>Excellent: ≤20% increase in maximum thickness using the most dense area on the follow-up vs baseline at both the 1- and 12-hour post infusion time points<br>Good: >20% but <35% increase in maximum thickness using the most dense area on the follow-up at +12h vs baseline<br><br>**Subdural hematoma:**<br>Excellent: ≤20% increase in maximum thickness at both the 1- and 12-hour post infusion assessments compared to baseline<br>Good: >20% but < 35% increase in maximum thickness at +12h compared to baseline<br><br>Hemostatic outcomes after 12 hours of AA administration
Culbreth et al,49 2019	Repeat CT scan -categorized as stable or worsening – no further details provided
Brown et al,47 2020	**Expansion**: >20% increase from pre-treatment hematoma volume or diameter; For IPH, volume was determined by the ABC/2 method<br>- Clinical efficacy was defined as lack of hematoma expansion
Barra et al,35 2020	**Excellent hemostasis** was defined as ≤ 20% increase in intracerebral hematoma volume, subarachnoid hemorrhage (SAH) thickness, or subdural hematoma (SDH) thickness.

© 2022 Chaudhary R et al. *JAMA Network Open.*

	**Good hemostasis** was defined as > 20% but ≤ 35% increase in intracerebral hematoma volume, SAH thickness, or SDH thickness. Poor hemostasis was defined as > 35% increase in intracerebral hematoma volume, SAH thickness, or SDH thickness.<br><br>- Repeat imaging within 24-hours of antithrombotic reversal
Coleman et al,36 2020	Only mortality data available, no data available to evaluate ICH hemostasis
Giovino et al,48 2020	Hemostatic efficacy on repeat head computed tomography (CT) after administration of andexanet alfa. The definition for hemostatic efficacy was adapted from the ANNEXA-4 trial[13], where an increase in hematoma expansion from baseline CT of ≤ 35% was defined as excellent or good and > 35% was considered poor efficacy
Ammar et al,37 2021	Stable head computed tomography (CT) scan at 6 and 24 h post-administration of AA or 4F-PCC, defined for IPH as no significant increase in volume (less than 6 mL or 33% of baseline volume) Stability was defined as a similar amount of blood from one scan to the next. For intraparenchymal hemorrhages, the volume of the hematoma was calculated using the ABC/2 volume estimation method. In IPH, a similar amount of blood was defined as a volume growth of less than 6 mL or 33% from baseline CT and adjudicated by the three experienced independent providers.
Stevens et al,39 2021	Difference in the achievement of excellent or good hemostasis within 12 h of andexanet alfa administration as compared to 4F-PCC. Hemostasis was defined based on the ANNEXA-4 study[13] definitions and classified as excellent, good, or poor. Excellent and good hemostasis were combined as "effective" hemostasis<br>Hematoma volume was calculated using the ABC/2 volume estimation method
Sobolewski et al,51 2021	Hematoma expansion on imaging – mean time between imaging was 12.2 hours (5:15 – 22:40) – no further data available
Benz et al,52 2022	Same definition as ANNEXA-4 trial[13]
Pham et al,40 2021	Excellent and Good as per ANNEXA- 4[13, 72]<br>CT scans 12-hr apart
Parsels et al,41 2022	Excellent or good hemostasis based on ANNEXA-4 study[13, 72]
Milioglou et al,42 2022	Brain imaging on presentation and repeat imaging within the first 24-or 48-h were assessed. Hemostasis effectiveness was evaluated by two physicians who reviewed separately all CT and/or MRI results before and after intervention (PCC or andexanet). Differences between brain hemorrhage volume estimates between the two physicians were resolved by discussion. No further information provided.
Vestal et al,44 2022	Hemostatic efficacy was determined by radiologists' interpretations of the diagnostic and first subsequent head scans. If radiologist's report stated an increase in hematoma volume, hemostatic efficacy was labeled as "No". If no progression was noted by the interpreting radiologist, hemostatic efficacy was labeled as "Yes". In terms of imaging, it was the standard institutional clinical practice to obtain a repeat scan six hours after the initial scan to assess progression of the hematoma.
**Idarucizumab**	
Pollack et al,12 2017	Maximum percentage reversal of the anticoagulant effect of dabigatran, determined at any point from the end of the first idarucizumab infusion until 4 hours after the end of the second infusion, with the percentage reversal assessed on the basis of the diluted thrombin time or the ecarin clotting time.<br><br>Complete reversal was defined as a decrease in the diluted thrombin time or ecarin clotting time to a normal level.<br><br>Brain imaging studies to evaluate for bleeding cessation were not mandated.
Sheikh-Taha et al,52 2019	Efficacy of idarucizumab using the International Society of Thrombosis and Hemostasis Scientific and Standardization Subcommittee (ISTH/SSC) criteria[72]
Singh et al,54 2020	Hemostasis not measured

© 2022 Chaudhary R et al. *JAMA Network Open.*

Kermer et al,55 2020	Information on hematoma growth with clinical deterioration (indicated by increase in NIHSS) with follow-up CT scan and comparison of hematoma volume at discretion of the attending physician. No further information provided
Yasaka et al,56 2020	ICH data unavailable for effective hemostasis.
**4-F PCC**	
Grandhi et al,24 2015	Progression of ICH was identified through review of subsequent head CTs and confirmed by radiology reports noting increased size of ICH on follow-up scans. No further information provided.
Majeed et al,25 2017	- Effectiveness assessment as per ISTH criteria[72]<br>- For ICH, follow-up CT within 24 hours, when available, was compared with the initial CT when available.<br>- Effective hemostasis is achieved when:<br>a. The hematoma volume is stable, or increased by <35% as compared with baseline volume as assessed by a CT scan within 12 h (time window of 6–24 h after the index CT)<br>b. No deterioration of the Extended Glasgow Outcome Scale (GOS-E) as assessed at 24 h in comparison with that at presentation.<br>c. By 48 h after the start of the initial management, there is no need for further infusion of hemostatic agents or coagulation factors, or transfusion of other blood products.<br>Annotation of effective hemostasis with good clinical outcome when:<br>d. No neurologic deterioration/dysfunction is present at discharge (at discharge can be replaced by 'at day = 30', whenever applicable) as assessed with any validated scoring system (e.g. GOS-E) as compared with that at presentation.<br><br>All of the above criteria had to be met for the therapy to be considered effective.
Gerner et al,8 2018	Hematoma enlargement defined as a relative parenchymal volume increase of >33% from initial to follow-up imaging.
Schulman et al,26 2018	**Good**: ≤20% increase in hematoma volume compared with baseline on repeat CT scan performed and/or any neurological improvement noted over the following 12 h or—if the patient was progressively deteriorating until the treatment with FEIBA—even a stabilization of the condition<br>**Moderate**: >20%, but ≤35% increase in hematoma volume compared with baseline on a repeat CT scan performed and/or minimal deterioration of neurological condition<br>**Poor/none**: >35% increase in hematoma volume compared with baseline on repeat CT scan performed at the 24-h time point and/or clear deterioration of the condition or death.
Tao et al,27 2018	Efficacy data for ICH not separated
Sheikh-Taha et al,28 2018	International Society of Thrombosis and Hemostasis Scientific and Standardization Subcommittee (ISTH/SSC) criteria[72]
Smith et al,29 2019	**Excellent**: ≤20% increase in hematoma volume compared to baseline on repeat CT scan performed at the 3- and 24-hour time points.<br>**Good:** >20%, but ≤35% increase in hematoma volume compared to baseline on a repeat CT scan performed at the 24-hour time point<br>**Poor/none**: >35% increase in hematoma volume compared to baseline on repeat CT scan performed at the 24 hour time point
Berger et al,30 2020	Hemostatic effectiveness within 24 hours of 4F-PCC administration. Hemostatic effectiveness was achieved if the first neuroimaging result within 24 hours of 4F-PCC administration showed no change or an improvement in hematoma volume
Zheng et al,31 2020	Re-evaluated by head CT within 8 hours of 4F-PCC administration – stable size. No other information was provided.
Korobey et al,32 2020	ANNEXA-4 criteria[13]
Castillo et al,33 2020	ANNEXA-4 criteria[13]
Lipari et al,34 2020	ANNEXA-4 criteria[13]

Smythe et al,38 2021	ANNEXA-4 criteria[13]
Pasciolla et al,45 2021	ANNEXA-4 criteria[13]<br>Excellent and good included in outcomes. Repeat CT head in 12 hours to assess hemostatic outcomes
Dev et al,43 2022	ANNEXA-4 criteria[13]

© 2022 Chaudhary R et al. *JAMA Network Open.*
